# Glanders: An ancient and emergent disease with no vaccine or treatment on site

**DOI:** 10.1371/journal.pntd.0013160

**Published:** 2025-06-11

**Authors:** Alfredo G. Torres

**Affiliations:** Department of Microbiology and Immunology, University of Texas Medical Branch, Galveston, Texas, United States of America; Institute of Continuing Medical Education of Ioannina, GREECE

## Abstract

Glanders is a highly contagious and potentially fatal zoonotic disease that primarily affects equines but can also infect humans. Caused by the bacterium *Burkholderia mallei*, the disease is characterized by nodular lesions in the lungs and ulcerations of the skin and mucous membranes. This review explores the historical significance of glanders, from its early recognition to its role as a biothreat agent. Additionally, it examines the global distribution and epidemiology of glanders, emphasizing its status as a re-emerging disease in certain regions. Finally, the clinical presentation, diagnosis, and host-immunity responses to *B. mallei* infection are discussed, with a focus on the ongoing efforts to develop a viable vaccine.

## Author summary

Glanders, a fatal zoonotic disease caused by *Burkholderia mallei*, affects equines and humans. This review covers its history, biothreat potential, global epidemiology, and re-emergence. It also explores clinical presentation, diagnosis, host immunity, and ongoing vaccine development efforts against *B. mallei* infection.

## Introduction

Glanders is a zoonotic infection caused by the Gram-negative bacterium *Burkholderia mallei*. This microorganism needs an animal host to survive because it is an obligate mammalian pathogen. The primary natural reservoir for *B. mallei* are members of the family Equidae (horses, mules, donkeys) [1,2], while humans are considered accidental hosts. Although *B. mallei* shares ~99% DNA sequence identity with the free-living opportunistic human pathogen *Burkholderia pseudomallei* [2], it has been proposed that *B. mallei* adapted to its mammalian host by reductive evolution of its genome [3]. *B. mallei* has a long history associated with natural and intentional infections, and is considered a biothreat agent. Despite the eradication efforts of the glanders disease from many areas of the globe, outbreaks in endemic areas have resurfaced the concern about this re-emerging pathogen.

### Historical view of *Burkholderia mallei* and glanders

**Fig 1:**
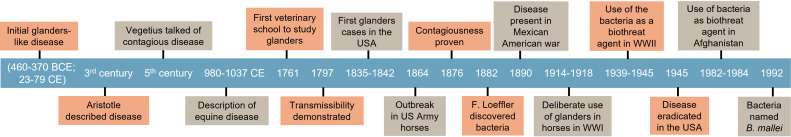
Timeline of the discovery of glanders disease and identification of *Burkholderia mallei.* The timeline highlights historical events associated with the recognition of glanders as horse disease, identification of the etiological agent, and its use as a biothreat agent. WWI, World War I; WWII, World War II. Figure created in BioRender. Torres, A. (2025).

The debate whether glanders was contagious remained deeply divided until Viborg demonstrated transmissibility in 1797 [4]. Later, in 1876, the disease came to be accepted as contagious after the pioneering work by Pierre Francois Olive Rayer, who inoculated a horse with pus from a groom that died of glanders—and the animal developed the disease (**Fig 1**) [5,6].

**Fig 2:**
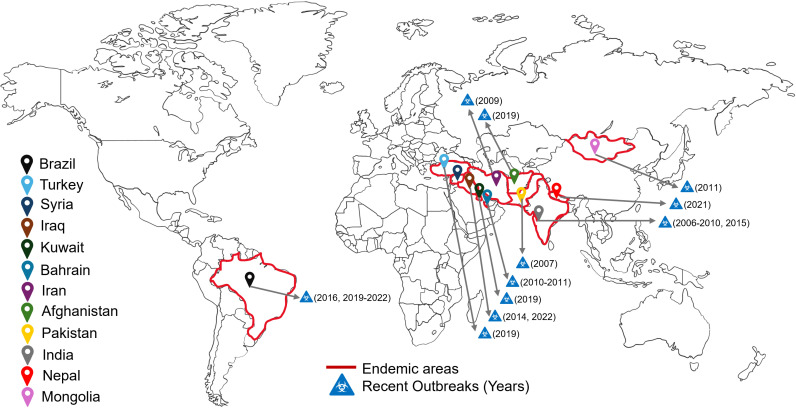
World map of glanders endemicity and recently reported outbreak areas. Countries in which glanders have been reported as an endemic disease (location pins and red outline). Recent outbreaks (biohazard symbol) with year of the last reported outbreak in parenthesis. Figure created in BioRender. Torres, A. (2025) and modified in Powerpoint.

The field of modern microbiology radically shifted in the late 19th century when Robert Koch outlined his postulates as guidelines to describe microbial disease in humans. It was this revolution that allowed Frederich Loeffler to isolate the glanders organism from the lung and spleen of an infected horse, thereby ending the debate on the etiological agent of glanders (**Fig 1**) [7,8]. The first extensive clinical study of glanders was conducted during the U.S. Civil War by two U.S. Confederate Army surgeons [4,5]. Since its initial isolation, *B. mallei* has been re-classified as a member of different genera, including *Bacillus*, *Corynebacterium*, *Mycobacterium*, *Loefflerell*a, *Pfeifferella*, *Malleomyces*, *Actinobacillus*, and *Pseudomonas* [1,4,8]. Most recently, in 1992, *B. mallei* was classified in the genus *Burkholderia* on the basis of 16S ribosomal DNA sequences, DNA–DNA homology, and physiological characteristics, such as cellular lipid and fatty acid composition, as well as phenotypic characteristics [9]. Extensive control programs by many countries including the United States of America (USA), Canada, and the United Kingdom, in the latter part of the 20th century led by increasing information about the pathology, epidemiology, and diagnostics allowed the eradication of the disease in these countries [1,2,7]. Today, there are few reported cases of glanders in humans; the last human infection in the USA was recorded in 2000. Nonetheless, glanders continues to occur in parts of Asia, South America, Northern Africa, and it is endemic in Iraq, Pakistan, India, Mongolia, and parts of Brazil [4,10]. Due to the number of recent outbreaks in the last 10–20 years (**Fig 2**), glanders has retained its classification as a re-emerging disease [1,2,4,11,12].

### Ancient and medieval periods

Glanders has a history that stretches back to antiquity, with the first recorded descriptions appearing in the writings of early Greek and Roman scholars [1,4,7,8]. The Greek physician Hippocrates (c. 460–370 BC) and Roman naturalist Pliny the Elder (23–79 AD) referred to a disease affecting horses that resembled glanders (Fig 1) [1,7,13]. Aristotle grouped glanders under the general description of diseases to infect both animals and humans (zoonotic diseases) and gave it the name ‘melis’ in Greek meaning “severe disease” (or mallus in Latin meaning, “malignant disease”) [4,5]. These early accounts, while not detailed by modern diagnostic standards, suggest the presence of glanders in ancient civilizations where horses played a crucial role in transportation, agriculture, and warfare. The first documented recognition of glanders as contagious disease was by the ancient Roman historian Vegetius in the fifth century, who recommended the separation of infected horses to prevent the spread of the disease [14].

In the medieval period, glanders became more widely recognized as a significant disease among horses. Veterinary manuscripts from the Islamic Golden Age, such as those by the Persian polymath Avicenna (980–1037 AD), provided descriptions of equine diseases that likely included glanders [15]. These texts were instrumental in disseminating knowledge about the disease throughout the known world, influencing veterinary practices in Europe, Asia, and the Middle East.

### Renaissance to the 19th century

The Renaissance period saw advancements in veterinary science, particularly in Europe, where the importance of maintaining healthy livestock was increasingly recognized. During this time, glanders was frequently documented in military horses, leading to its study by veterinarians serving in various armies [16,17]. The disease’s impact was profound, often resulting in substantial losses of valuable cavalry horses.

In the 18th and 19th centuries, glanders emerged as a critical issue for veterinary medicine, prompting more systematic investigations. The founding of veterinary schools, such as the École Nationale Vétérinaire de Lyon in 1761, facilitated the scientific study of glanders (Fig 1) [18]. Researchers began to understand the clinical features and transmission patterns of the disease, laying the groundwork for modern bacteriology.

### Discovery of *B. mallei*

A breakthrough occurred in the late 19th century with the identification of the causative agent of glanders. In 1882, the German microbiologist Friedrich Loeffler successfully isolated the bacterium *B. mallei* [19]. Loeffler’s work, conducted alongside Robert Koch, marked a significant advancement in the understanding of infectious diseases (Fig 1). Their research confirmed that glanders was caused by a specific pathogen, paving the way for diagnostic and therapeutic developments [8].

### Twentieth century

The 20th century witnessed both the decline and resurgence of glanders, influenced by changes in horse populations and advancements in veterinary care [2,8]. By the mid-20th century, stringent control measures and the advent of antibiotics led to the near eradication of glanders in many regions. However, the disease’s potential as a bioweapon became a concern during both World Wars [20]. Therefore, the biowarfare capacity of glanders is discussed below.

### Modern era and re-emergence

In recent decades, glanders has re-emerged as a significant concern in certain parts of the world, particularly in regions with less stringent veterinary infrastructure [2,4]. Outbreaks have been reported in Asia, the Middle East, and South America, prompting renewed attention to the disease (Fig 2) [2]. Factors contributing to its resurgence include increased movement of horses, lapses in biosecurity, and the bacterium’s intrinsic resistance to multiple antibiotics [4].

Research efforts focus on developing more effective diagnostics, vaccines, and treatments to combat glanders [21]. International organizations, such as the World Organization for Animal Health [22,23] and the EFSA Panel on Animal Health and Welfare [24], play a crucial role in coordinating global efforts to monitor and control the disease. The limited reported number of human glanders cases underscores the importance of continuous vigilance in the field of veterinary and human medicine.

### *B. mallei* as a biothreat agent

The historical use of *Burkholderia mallei* as a biothreat agent dates back to the 4th century during the reign of Constantine the Great [4]. Since glanders primarily affects horses—an essential means of transportation throughout history—the disease imposed a significant economic burden [5]. Notably, glanders afflicted Crusaders by infecting their horses [4,5]. Recognizing its dangers, Louis XV established the first veterinary school in Lyons to study glanders’ impact on the French cavalry [5]. The first recorded case of glanders in the United States occurred during the American Revolution among British cavalry horses, but it gained prominence during the Second Seminole War (1835–1842) [5]. While no documented evidence confirms intentional use, the U.S. Bureau of Animal Industry admitted in 1890 that glanders may have been introduced into Mexico during the Mexican–American War through diseased U.S. Army horses (Fig 1). Glanders was extensively documented during the American Civil War, where Confederate forces reportedly left infected horses behind after battles, leading to widespread infections among horses, mules, and civilians [5]. One of the most devastating outbreaks occurred at the Federal Army’s largest horse supply depot, Giesboro, in 1864. Infected horses sold to the Federal Army resulted in mass exposure, with estimates suggesting nearly a quarter of a million animals were affected, and a record 188 deaths occurred in a single day [5]. After the Civil War, many surviving horses from infected depots were either sold or distributed to the public, though records of their eventual fate remain scarce [5].

The use of *B. mallei* in warfare persisted into the early 20th century, particularly during World War I (WWI). Over 58,000 French army horses were infected, primarily due to transmission from captured Russian horses [1,25–27]. WWI also saw the first documented intentional use of *B. mallei* as a bioweapon when German agents deliberately contaminated shipments of allied horses, mules, cattle, and sheep with microbial cultures (Fig 1) [25]. The use of *B. mallei* as a bioweapon continued during World War II, when Japanese forces deployed the bacterium against horses, civilians, and prisoners of war at the Píngfáng military base in Manchukuo, a Japanese-controlled puppet state in China (Fig 1) [1,28]. Reports suggest its use persisted into the late 20th century, with claims that the Soviet Union intentionally infected Mujaheddin horses during the Afghan Wfisar between 1982 and 1984 [1,29]. Since that time, no further documented cases of *B. mallei* being used as a bioweapon have emerged. These events underscored the dual-use nature of glanders research, necessitating careful oversight and regulation [7,8,30]. Therefore, due to its ability to infect humans, lack of effective treatments, antibiotic resistance, and potential for weaponization, *B. mallei* was classified as a Tier 1 Select Agent by the U.S. Department of Health and Human Services and the Centers for Disease Control and Prevention in 2000 [7,24,26]

## Epidemiology

### The host

Solipeds, or firm-hoofed animals as horses, asses, and mules, are susceptible to glanders disease, caused by *B. mallei*. Interestingly, acute forms of the infection occur most frequently in donkeys and mules, with high fever and respiratory signs, whereas horses generally present a more chronic course and may survive for several years, especially in endemic areas [1,2]. Interestingly, the name glanders originated from the lymphangitis and lymphadenopathy (glands) that are associated with disease in horses. In the case of cutaneous manifestations, the disease is called Farcy. Although less susceptible, humans, and occasionally felids, camels, bears, wolves, and dogs are susceptible to infections. Other carnivores may become infected by eating infected meat; however, cattle and pigs are resistant [1,2].

### Sources of infection and transmission

Transmission of *B. mallei* from horses or other solipeds to humans appears to be uncommon, even when frequent and close contact with infected animals is occurring [30]. Despite the low incidence of animal-to-human transmission, occupational exposure of the animal handlers remains a key risk factor, particularly veterinarians, soldiers, slaughterhouse personnel, farmers, and other horse-handling professions. Human-to-human transmission is also rare. However, it may occur during occupational exposure in medical practice or at autopsies [30]. In the case of laboratory workers, they have rarely been infected; however, close contact with high concentrations of virulent bacteria might put them at considerable risk for infection. In the case of animal-to-animal transmission, the most common source of infection appears to be ingestion of contaminated food or water likely via discharges from the respiratory tract or ulcerated skin lesions from carrier animals [30]. Animal density and proximity favor spread as well as stress-related host factors. Although the disease could be technically treated with antibiotics, this is not permitted under current regulations [22–24]. Therefore, to stop the bacteria from becoming established and to avoid animal-to-human transfer, affected animals should be euthanized.

### Occurrence

Early in the 20th century, glanders disease was still widely present worldwide; however, the effective use of veterinary interventions (large-scale culling) and national control programs initiated between the 40s and 50s had significantly reduced the prevalence of this disease. Irrespectively of the implementation of these interventions, glanders continues to be reported in Brazil, India, Iran, Iraq, Pakistan, Turkey, and the United Arab Emirates (UAE) and it is thought to be endemic in various areas of Asia, Africa, and South America [4,8]. In such countries, economic and cultural circumstances may hinder culling of asymptomatic animals, enabling the persistence of glanders disease.

In recent years, several outbreaks occurred in horse populations in Asia, including Western Asia (Afghanistan, Kuwait, Iran, Iraq, Pakistan, Syria), Africa, and South America (Brazil). Further, recent rise of glanders cases in horses, in combination with worldwide horse trading, results in the potential for the disease to be re-establish in countries in which it has been previously eradicated (glanders is now considered a re-emerging disease) [4], and posing new risks for human infections.

## Selected glanders outbreaks in endemic areas

### Glanders in Bahrain

In April 2010, a large outbreak of glanders was reported in Bahrain, an archipelago of 36 islands in the Persian Gulf off the eastern coast of Saudi Arabia, and home to about 6,500 horses. Bahrain was considered a glanders-free country until horses imported from Syria via Kuwait were suspected of introducing glanders [31]. By September 2010, the outbreak was considered resolved. However, in January 2011 the disease re-occurred in the same region of the country, and, at the end of the investigation, 50 horses and 1 camel tested positive, with the bacteria being isolated from the camel and 8 horses (Fig 2).

Genotypic and comparative analysis from the bacterial strains isolated in 2010 to those of a prior *B. mallei* outbreak in the UAE in 2004, indicated that the samples from the outbreak in Bahrain were separated into two distinct clusters, suggesting that two independent but simultaneous strain introductions took placed and caused the outbreak [31]. Multilocus variable number tandem repeat analysis of the *B. mallei* strain isolated from a diseased camel in Bahrain was used to confirm and results revealed close genetic proximity to UAE strain Dubai 7, confirming that glanders disease in this animal was the result of the outbreak but caused by a second strain [10].

### Glanders in Brazil

Brazil is considered a glanders endemic country and the disease continues to pose a significant threat to equids and public health, where it is considered a re-emerging disease. Historically, foci of glanders occurred with more frequency in the north and northeast of the country; for example, in equids of the “Zona da Mata” in the states of Pernambuco and Alagoas [32]. The prevalence of glanders disease in Brazil was in the spotlight during the 2016 Olympic Games at Rio (Fig 2) [33]. At the end of July 2015, it was confirmed that at least 17 horses were diagnosed with glanders, and all the animals were quarantined or euthanized in Cananea Island, near Sao Paulo. This information was relevant because it was believed that some of the horses at Cananea Island came from the Deodoro military complex, a place housing the Army Equitation School, which was located close (0.35 mi) to the Olympic Equestrian Centre. The Ministry of Health confirmed that despite the threat of glanders near Rio de Janeiro, the situation was not a threat to the health security of Olympic events; and therefore, all the equestrian events continued without an incident [34].

The epidemiological situation in Brazil remains inadequately understood. A study in Pará estimated a 1.68% prevalence of infected properties and identified the introduction of new animals as a key risk factor. Despite the low overall prevalence, current control measures have not significantly impacted the endemic balance of glanders [35]. A spatial and temporal analysis of glanders cases in Piauí between 2015 and 2022 revealed high-incidence areas, particularly in Campo Maior, Teresina, and Altos, with a significant cluster emerging from 2019 to 2022. Glanders remains well-localized but have the potential to spread across borders. Nationwide, molecular detection of *B. mallei* in seropositive equids across all five Brazilian regions demonstrates the disease’s expansion and its persistence in asymptomatic animals. Environmental elimination of *B. mallei* is also a concern, as microbiological detection in nasal and palate swabs suggests the potential for ongoing transmission [36]. Further, the mandatory obligation of owners to sacrifice disease-positive animals without compensation results in severe economic losses and trade restrictions [37]. Therefore, a full economic impact analysis of glanders disease is needed to support further research with this pathogen.

More recent studies in Brazil examined clinical manifestations, pathological findings, and diagnostic methods in naturally infected equids, highlighting the importance of combining histopathology, bacterial culture, and PCR for accurate detection. Among 16 infected horses and one fetus from three outbreaks, 37.5% showed no clinical signs, underscoring the role of asymptomatic carriers in disease transmission. PCR confirmed *B. mallei* in all tested animals, with bacterial isolation occurring in 8.2% of samples [38]. Given its economic and epidemiological implications, Brazil must reassess its strategies, incorporating both public and private efforts to contain the disease. Mandatory reporting by the World Organization for Animal Health emphasizes the global significance of glanders, reinforcing the need for continued surveillance, improved diagnostics, and effective containment strategies to prevent further spread.

### Glanders in India

In this country, the disease has been detected among horses, donkey, and mules, but restricted to certain geographical pockets with sporadic cases detected in the 80s–90s. Because animals are used for transport, there is a constant, perceptible threat to the re-emergence of this disease in equines, due to work stress and cross-border exposure. Monitoring activities of the disease resulted in the identification of outbreaks occurring in different Indian States from 2006 to 2010 (Fig 2) [39] and culminating with a major glanders outbreak among equines in the state of Maharashtra [12]. During these outbreaks, a total of 164 equids were found positive and the infected animals were euthanized, and control measures were implemented [39,40].

The potential for human exposure to the disease has been of significant concern due to the detection of glanders among animals carrying pilgrims to the Holy Cave Shrine of Mata Vaishno Devi Ji back and forth from Ban Ganga to Bhawan, raising concerns over the safety of the pilgrims as well as people living in the holy town of Katra, the base camp where the shrine is located (Fig 2) [41]. During the pilgrimage, large numbers of working animals are used (estimates indicated approx. 5,000 mules) to carry the pilgrims, which indicates that one single case can quickly spread to other animals and humans. Overall, from 1,704 blood samples taken from animals, 17 tested positive for the disease, and all the animals were euthanized [41]. Immunological studies in 151 glanders-positive equids revealed strong IgG responses, particularly against the Hcp1 antigen, while cytokine profiling indicated elevated levels of IL-1β, MCP-1, IL-17, IL-6, IFN-γ, and TNF-α, highlighting their role in immune defense [42]. These findings suggest Hcp1 as a key target for immune response and emphasize the need for further research on memory cell responses to develop effective vaccines against *B. mallei*.

Recently, the re-emergence of the disease has been detected since 2006, with outbreaks reported in fourteen states. A survey of 165 veterinarians, para-veterinarians, and physicians from glanders-affected regions revealed significant knowledge gaps, with 40.3% unaware of government regulations and 22% unaware of disease transmission [43]. Many respondents (68.4%) noted reluctance among equine keepers to report clinical symptoms. Challenges in disease control included lack of cooperation from authorities (33.9%), financial constraints (31%), and administrative (28.4%) and technical (27.8%) limitations. Improved training and intersectoral coordination are needed for effective control [43]. Overall, emergence or re-emergence of the pathogen is an example of the need of implementing control actions to prevent dissemination because the disease can pose a significant threat to Indian residents and visitors.

## Clinical presentation

In humans, glanders is primarily transmitted through direct invasion of broken skin, inhalation, or mucosal contact [2,30]. Occupational exposure often occurs through exposed skin on the hands, arms, neck, and face. Glanders presents in multiple forms, including chronic, disseminated, pulmonary, and septicemic infections, largely depending on the route of exposure [2,30]. Localized infections typically cause abscesses that can ulcerate and drain for extended periods but may spread, leading to more severe systemic involvement. Cutaneous glanders appear as papular lesions that may ulcerate, causing pain, swelling, and lymphatic inflammation. Pulmonary glanders can progress to pneumonia, pleuritis, and pulmonary abscesses, with symptoms including respiratory distress. Disseminated infections result in septicemia and organ abscesses, primarily affecting the liver, spleen, and kidneys. Glanders incubation periods vary; acute cases manifest within 1–14 days, while chronic cases take up to 12 weeks [2,30]. Localized infections may develop within days, while pulmonary disease and septicemia progress rapidly, with untreated pneumonic glanders proving fatal within 10–30 days. Notably, some patients experience temporary symptom relief before a second wave of illness, which may be misinterpreted as recovery [30]. This pattern underscores the need for continuous treatment and monitoring to prevent misdiagnosis and ensure complete eradication of the infection.

In equines, glanders manifests in three primary forms—pulmonary, nasal, and cutaneous—often occurring simultaneously [1,4]. The disease can be acute, subacute, or chronic, with pulmonary involvement being most common. Acute glanders primarily affects donkeys and mules, leading to rapid deterioration and death within days to weeks. Horses are more prone to chronic glanders, which can persist for months or years, occasionally improving before worsening again. Chronic cases are usually fatal, though some animals may recover and become lifelong carriers. Risk factors include age, overcrowding, poor climate conditions, and contact with infected animals [1,4].

Acute glanders begins with fever, depression, and nasal discharge, progressing to respiratory distress and pneumonia, often resulting in death. Chronic glanders can be insidious, with intermittent fever, coughing, and progressive weakness [1]. Severe cases lead to nasal ulcers, enlarged lymph nodes, joint swelling, and testicular inflammation. Cutaneous glanders, or farcy, presents as nodular lymphangitis with ulcerating abscesses along lymphatic vessels. These lesions can persist, causing chronic discharge and debilitating weakness. In severe cases, glanders can spread to internal organs or the nervous system, exacerbating symptoms [1]. The disease is highly contagious and fatal, making early detection and management essential in affected equine populations.

## Diagnosis

Diagnosing glanders in equines requires a combination of clinical examination, serological tests, bacterial culture, and molecular diagnostics [1]. In the case of clinical examination, the observation of characteristic symptoms such as nasal ulcers, mucopurulent discharge, lymphadenopathy, pulmonary distress, and cutaneous nodules (farcy) is indicative of the disease. In the case of chronic cases, they may show intermittent fever, weight loss, and joint swelling. The commonly used mallein test (delayed-type hypersensitivity test), consists of injecting intradermally or into the lower eyelid of the animal, a purified protein derivative of *B. mallei* (mallein), and in the case of a positive reaction, swelling, purulent discharge, and conjunctivitis occurs within 24–48 h [1].

Other available tests include the use of serology, such as the complement fixation test, which is the official confirmatory test for glanders because it detects antibodies [1]. Sera can also be used for ELISA, which are more sensitive, and Western Blot is used for confirmatory diagnosis. In the case of bacteriology, these tests include sampling from nasal discharge, ulcers, or tissue are culturing on selective media. This approach is used to identify *B. mallei,* but it is time-consuming and requires biosafety precautions. In the case of molecular diagnosis, PCR is used to detect *B. mallei* DNA in clinical samples and is useful for early detection [1]. Finally, histopathology of tissue biopsies may help to identify granulomatous inflammation and radiography, or ultrasound may detect lung abscesses and internal organ involvement.

The definitive diagnosis of human glanders requires isolating and identifying *B. mallei* [30]. Radiology can reveal abscesses in organs like the lungs, liver, and spleen, but these are not specific to glanders. No validated *in vitro* diagnostic test exists, but experimental serological tests, such as agglutination, complement fixation, and PCR-based methods, have been used. The indirect hemagglutination assay, used for melioidosis, can also detect glanders, though it lacks specificity due to cross-reactivity with other *Burkholderia* species [30]. The mallein test is used in veterinary diagnostics but not for humans. Overall, it is evident that more specific and sensitive diagnostic methods are needed because the available test were developed in the past century [1,4] and no modern test are on the market today.

## Treatment

The treatment of glanders in animals is not recommended in most cases due to the high risk of zoonotic transmission, the difficulty in completely eradicating the infection, and the potential for relapse [4]. Instead, the international veterinary regulations called for euthanasia of infected animals to prevent the spread of the disease [24]. All animals in contact with the infected animal are quarantined, tested, and reported since glanders is a notifiable disease in most countries. However, in rare and highly controlled research or military settings, treatment with long-term antibiotics (e.g., doxycycline, ceftazidime, or imipenem) may be explored, but not in routine veterinary practice [44,45].

In the case of human glanders, it requires aggressive and prolonged antibiotic therapy. Because human glanders cases are rare and *B. mallei* is inherently resistant to many antibiotics, treatment protocols are based on susceptibility data and experience from melioidosis patients, disease caused by *B. pseudomallei* [46]. The general treatment includes an initial intensive therapy phase (10–14 days) with the use of intravenous ceftazidime, imipenem, or meropenem; followed by an eradication phase (3–6 months) with doxycycline or trimethoprim-sulfamethoxazole [47].

## Host immunity to *B. mallei* infection

### Humoral and cellular responses to *B. mallei* infection

Host immune responses to *B. mallei* involve both innate and adaptive immunity, though the exact mechanisms are still being studied. It is known that the immune system recognizes *B. mallei* through PRRs such as Toll-like receptor (TLR) 4, triggering inflammatory responses [48,49]. *B. mallei* proteins manipulate various cellular processes, including host ubiquitination pathways, phagosomal escape, and actin-cytoskeleton rearrangement. Proinflammatory cytokines such as TNF-α, IL-1β, and IL-6, play key roles in recruiting immune cells to the infection site; however, the pathogen has mechanisms to evade or survive within macrophages, leading to persistent infection [48,49].

As an intracellular pathogen, humoral and cell-mediated immunity, particularly the role of CD4^+^ and CD8^+^ T cells, is postulated to be crucial for bacterial maintenance and latent infections. Interestingly, vaccine studies have demonstrated that immunization elicited a robust humoral immune response by production of *B*. *mallei*-specific total IgG, as well as IgG-specific isotypes. Re-stimulation of splenocytes from vaccinated mice with *B*. *mallei* whole cell lysates has also indicated that the cellular immune response induced IFN-γ and other inflammatory cytokines production. Therefore, the data suggest that CD4^+^ and CD8^+^ T cells responses may be mostly dispensable at the time of *B. mallei* exposure but may be critical at the time of vaccination, when B cell memory development and isotype class switching occur [50].

## Vaccine development

Currently, no licensed vaccine exists for either human or animal use against glanders [51–53]. However, various vaccine platforms targeting *B. mallei* have demonstrated protection against acute disease, mainly in small animal models of infection [52,53]. Despite these advancements, most vaccine candidates fail to confer complete protection against chronic glanders across different exposure routes, except at very low exposure doses [52,53]. Additionally, many vaccine approaches rely on immunization through the same route as infection to achieve optimal protection [51,52,54].

### Whole-cell vaccines

#### Live-attenuated vaccines.

These types of vaccines are the most promising candidates for glanders prevention, offering rapid, broad, and long-lasting protection without the need for adjuvants [51]. However, their use in humans raises safety concerns due to the potential for pathogen reversion and adverse effects, particularly in immunocompromised individuals [11,51,53,55]. These risks may be mitigated through genetic modifications that introduce multiple mutations or deletions to prevent reversion and reduce host persistence [51,53]. Several mutagenesis approaches have been explored to create live-attenuated *B. mallei* strains, including Iron transport system deletion (*tonB*) [56], Endoprotease mutation (*ctpA*) [57], Quorum sensing disruption (*bmal3*) [58], Amino acid biosynthesis deletion (*ilvI*) [59]. These mutant strains have been tested in murine models via inhalational, intranasal, or systemic exposure, with varying degrees of attenuation and protective efficacy [56,57,60]. For example: the *B. mallei* Δ*ilvI* strain provided short-term resistance against both high and low aerosol exposure doses, with 25%–50% of mice surviving up to one month post-infection [59]. Mice vaccinated with the Δ*bmal3* strain exhibited 30% survival at 11 days post-infection following aerosol exposure [60]. The *B. mallei* Δ*ctpA* mutant conferred 75% survival in a systemic glanders model at 15 days post-infection [57]. One of the most promising vaccine candidates, *B. mallei* strain CLH001, features deletions in both *tonB* (iron transport) and *hcp1* (hemolysin coregulated protein 1) [11]. This strain provided complete protection for up to 21 days post-exposure, preventing liver and lung colonization. However, at higher exposure doses, bacteria were still detected in the spleen [11]. While many live-attenuated vaccines provide partial to full protection against acute infection through different exposure routes, none have demonstrated complete protection against chronic glanders [21]. Interestingly, the *B. mallei* Δ*tonB* mutant remains the most effective candidate against both glanders and melioidosis in lethal intranasal challenge models [53,56]. Further studies are necessary to assess safety and efficacy in human populations.

#### Killed vaccines.

These vaccines present an alternative to live-attenuated vaccines, though they often fail to elicit strong cell-mediated immunity, which is critical for bacterial clearance [51,52]. The use of adjuvants has been explored to enhance immune responses while maintaining safety [52]. Heat-killed *B. mallei* vaccines induced mixed Th1/Th2 immune responses in mice, with a 40% mean survival rate against a lethal challenge of ~20 LD_50_ [61,62]. Irradiated vaccine formulations—including heat-irradiated, irradiation-inactivated, and capsule-mutant-inactivated strains—administered subcutaneously conferred 80%–100% protection at 21 days post-infection with low-dose exposure [62]. However, these formulations failed to protect against high-dose exposure [51]. Incorporating IL-12 into subcutaneously administered irradiated vaccines improved protection by up to 60%, likely due to its role in activating IFN-γ-producing T cells and enhancing Th1 responses [63]. A formalin-inactivated vaccine developed in Russia using wild-type *B. mallei* (strain 11) conferred 70% protection in guinea pigs when adjuvanted with aluminum hydroxide [55], This vaccine protected 70% of animals when adjuvanted with aluminum hydroxide in guinea pigs against *B. mallei* [55]. In humans, a single subcutaneous injection of this vaccine at 4 × 10⁹ CFU elicited detectable antibodies in 27.3% of individuals for up to 1-year post-vaccination [55].

#### Subunit vaccines.

These vaccines are a safer alternative to whole-cell vaccines, though their efficacy varies [7,51,52]. They primarily induce Th2-biased immune responses, which are less effective against intracellular bacteria like *B. mallei* [51,52]. However, polyvalent subunit vaccines combining multiple antigens may enhance protection across different strains and exposure routes [51,64,65].

#### Proteins subunit vaccines.

Most subunit vaccines use single proteins derived from key *B. mallei* virulence factors [51]. Studies have shown that: Mice vaccinated with *B. mallei* proteins Hcp1, BimA, BopA, or *B. pseudomallei* LolC achieved survival rates between 75% and 100% [66]. The highest efficacy was observed in mice immunized with BopA and BimA, which led to bacterial clearance from the lungs, although not from the spleen. These mice exhibited 100% survival at 21 days post-infection with 2 LD50 of wild-type *B. mallei* [66]. The BopA protein remains the most promising subunit vaccine candidate for cross-protection against both glanders and melioidosis [66].

#### Nanovaccines.

Gold nanoparticle (AuNP) vaccines have being explored as a promising approach for protecting against glanders by delivering protein subunits conjugated to *Burkholderia thailandensis* LPS [67]. These vaccines utilize gold nanoparticles as carriers to enhance immune responses by improving antigen delivery and stability. For example, the flagellin protein (FliC) conjugated to AuNP was tested in murine and rhesus monkey models. Mice challenged with wild-type *B. mallei* demonstrated 80%–100% survival against intranasal exposure (~3.5 LD50) at 21 days post-infection [67,68]. Increased protection was also observed in rhesus monkeys receiving subcutaneous immunization [67].

More recently, AuNP-based nanovaccines have been modified to enhance immune responses and develop multivalent vaccines. Investigators synthesized a nano-glycoconjugate vaccine by coupling six highly immunogenic antigens and *B. thailandensis* LPS to AuNPs [69]. Mice immunized intranasally with an optimized formulation (OmpW, OpcP, Hemagglutinin, and LPS) showed complete protection against lethal *Burkholderia mallei* infection. The nanovaccine elicited strong, sustained antibody responses, improving bacterial adherence inhibition and opsonophagocytic activity [69]. Overall, the potential advantages of these nanovaccines include increased antigen uptake, prolonged immune activation, and reduced side effects compared to traditional vaccines.

## Conclusion

*B. mallei* is a mammal-adapted bacterium that has been reclassified multiple times across different genera since its discovery. It remains a potential biothreat and is associated with both military and endemic cases. Recent outbreaks have led to its classification as a re-emerging pathogen, particularly in endemic regions. Despite this resurgence, *B. mallei* is highly lethal to both humans and equids. These characteristics underscore the urgent need for effective diagnostics, vaccines, and treatments. However, limited understanding of glanders pathogenesis has hindered the development of effective countermeasures against this re-emerging threat.
